# Sero—salivary detection of *H. pylori* immunoglobulins and parasitic infection among healthcare individuals suffering from gastrointestinal disorders with correlation to personal hygiene

**DOI:** 10.1186/s13099-025-00688-2

**Published:** 2025-04-10

**Authors:** Faika Hassanein, Mohamed S. Abdel-Latif, Amany I. Shehata

**Affiliations:** 1 Department of Microbiology & Immunology, Faculty of Dentistry, Pharos University in Alexandria, Alexandria, Egypt; 2 Department of Medical Laboratory Technology, Faculty of Applied Health Sciences Technology, Pharos University in Alexandria, Alexandria, Egypt; 3 Department of Tropical Health, High Institute of Public Health, Alexandria University, Alexandria, Egypt

**Keywords:** Saliva, *H. pylori*, Parasitic infection, Healthcare individuals, Protective measures, Personal hygiene, Immunoglobulins

## Abstract

**Background:**

Gastrointestinal microbial infections among healthcare individuals (HCIs) are common due to several risk factors, including poor personal hygiene and socio-economic lifestyle.

**Objectives:**

This is the first cross-sectional study that stratifies HCIs to correlate personal hygiene and socio-economic lifestyle with gastrointestinal microbial infections. Additionally, it compares serum and saliva levels of *H. pylori*-IgG and IgA to assess the potential of saliva as a non-invasive alternative to serum.

**Methods:**

Based on Fisher’s formula, 200 HCIs suffering from gastritis—including hospital workers, employees, nursing students, nurses, and doctors—were enrolled. Blood, saliva, and stool samples were collected for microbial infection investigations. Personal hygiene and socio-economic factors were scored based on WHO guidelines. Parasitic infections were identified microscopically, while *H. pylori* antigen and antibodies were detected via ELISA, with diagnostic significance determined by ROC curve analysis.

**Results:**

A high prevalence of intestinal microbial infections was observed among HCIs. *Blastocystis spp.* was the most common pathogen (72%), followed by *Cryptosporidium spp.* (59.5%). Cases of single, double, and multiple infections were detected. *H. pylori* antigen was present in 36 (18%) cases, often as a co-infection with intestinal parasites. Infection rates were highest among workers and nurses (100%), followed by employees (97.4%) and nursing students (81.7%), with doctors having the lowest rate (50%). Poor personal hygiene and socio-economic lifestyle were directly linked to increased infection risk. Additionally, *H. pylori*-IgG was positive in 14 cases and negative in 186 cases, while *H. pylori*-IgA was positive in 2 cases and negative in 198 cases in both serum and saliva. These findings indicate consistency between serum and saliva levels of *H. pylori* immunoglobulins.

**Conclusions:**

Poor personal hygiene and socio-economic lifestyle significantly increase the risk of gastrointestinal microbial infections among HCIs. Salivary immunoglobulins show consistency with serum levels, suggesting saliva as a viable non-invasive alternative for detecting *H. pylori* infection.

## Introduction

Gastrointestinal diseases are prevalent in tropical countries and often present with symptoms such as diarrhea, abdominal pain, abdominal distention, gastrointestinal bleeding, intestinal obstruction, malabsorption, or malnutrition. Special attention is paid to illnesses more common in the tropics, including duodenal ulcer, gastrointestinal infections, tropical enteropathy, and *Helicobacter* infection [1, 2].

Enteric parasitosis (EP) constitutes a major public health issue that blights the lives of billions of people worldwide [3]. Globally, 3.5 billion individuals are affected; 450 million are symptomatic, and more than 200,000 deaths are reported annually [4, 5]. For instance, developing countries in Sub-Saharan Africa bear a higher burden of intestinal parasites than developed nations which are with lower burden [6]. Enteric parasitosis constitutes an infection rate higher than 50% in Sub-Saharan Africa (SSA) due to several risk factors including poor socio-economic conditions, inadequate access to clean water, lack of proper sanitation, limited healthcare facilities, low community awareness, and unfavorable climatic and environmental conditions [7–10].

In Egypt, the prevalence of intestinal parasites is 61% among individuals with diarrhea, while the prevalence of *H. pylori* reaches up to 70% in dyspeptic patients [11, 12].

*Helicobacter pylori* is a widely recognized stomach pathogen that specifically infects humans, affecting over half of the global population. This bacterial infection can lead to various gastrointestinal issues such as chronic gastritis, peptic ulcers, and potentially cancer [13, 14]. The most common route of *H. pylori* infection is either oral or feco-oral contact. Its prevalence remains high in most developing countries, typically linked to socioeconomic status and hygiene standards. Global and regional *H. pylori* prevalence have not been systematically reported until now [13].

Co-infections with intestinal parasites and *H. pylori* are common gastrointestinal disorders that affect individuals worldwide, causing a significant burden on public health. Both infections are associated with a range of symptoms, including abdominal pain, diarrhea, and nausea, and can lead to chronic health complications if left untreated. There is a strong correlation between intestinal parasites and *H. pylori* regarding socioeconomic style despite having similar routes for transmission. *H. pylori* acts as a synergistic element for intestinal parasites and bacteria to easily invade the stomach acid barriers by the production of urease; and also this synergism has a clinical significance in dyspepsia [15]. Understanding the immune response to these infections is crucial for developing effective diagnostic and treatment strategies [16].

Serum immunoglobulins’ response to *H. pylori* infection is an important determinant of gastric mucosal damage. IgA is the predominant immunoglobulin in mucosal secretions of the gastrointestinal tract. Elevated levels of *H. pylori* IgA may indicate an active or recent infection. IgG is an important marker for detecting past exposure to *H. pylori*. IgA and IgG type of antibodies may remain high for months or years unless the infection is treated. After eradication of *H. pylori*, IgA levels decrease but IgG does not disappear [17, 18]. Salivary IgG is secreted during the serum immune response, and the levels parallel that of circulatory IgG levels. Also, it has been shown that the salivary *H. pylori*-IgG test revealed a high accuracy, sensitivity and a high negative predictive value. Moreover, saliva is superior to serum in terms of easy to collect, handle and store, with reduced risk of blood-borne infection. Thus, salivary IgG test has the privilege that makes it more feasible than serum IgG test [19]. The accuracy of sero-salivary immunoglobulines detection of *H. pylori* infection varies according to the antigens provided by the commercial kit and the antigenic composition of specific *H. pylori* strain in a specific population in particular geographical area [20].

Healthcare individuals providing patient care, who work in research, hospitals and clinical laboratories, are at risk of becoming infected with microorganisms through accidental exposures. Similarly, healthcare individuals with parasitic infections can transmit these infections to patients, especially those in critical care who are highly susceptible due to their compromised immune state and also infect other workers [21, 22].

Compared to senior registered nurses, nursing students are younger, inexperienced, and lack skills and professional knowledge about protective measures, making them more vulnerable to occupational injuries [23]. Additionally, they may not have enough background knowledge to recognize the risks posed by patients or understand standard infection control principles [24].

The Centers for Disease Control and Prevention states that inadequate hand washing by employees can make hands the primary means of transmitting enteric pathogens [25]. The clinical presentations of infectious gastroenteritis vary widely and can appear quickly. The incubation period for common microbial foodborne illnesses, including *Campylobacter*, *Cryptosporidium*, *Cyclospora*, and *Giardia*, ranges from 1 to 14 days, making it difficult to identify the food involved in transmission without microbiological diagnosis [26]. These microorganisms can spread easily because of low infectious dose pathogens (< 500 viable organisms) [27].

Consequently, this study was designed to be the first cross sectional study that stratified the healthcare individuals into; workers, employees, nursing students, nurses, and doctors to correlate personal hygiene and socio-economic lifestyle with gastrointestinal microbial infections caused either by parasites or *H. pylori*. Meanwhile, this study compared the levels of *H. pylori* antibodies (IgG and IgA) in serum and saliva samples trying to find a surrogate non-invasive method for laboratory detection of *H. pylori* antibodies.

## Materials and methods

Ethical approval for the study was obtained from the local ethical committee of Pharos University in Alexandria (ID: PUA-02–2023-8–27-3–128). Also, a written informed consent was obtained from all recruited individuals for this study.

### Study design and participants

A cross-sectional study was carried out in the outpatient clinics at Smouha University Emergency Hospital in Alexandria Governorate, Egypt. Based on Fisher’s formula, 200 healthcare individuals (HCIs) (70 males and 130 females) suffering from gastritis and aged from 18 to 59 years old with mean (30.63 ± 11.55) were enrolled in the present study from September 2023 to February 2024. The HCIs included hospital keeping workers, employees, nursing students (under training), nurses, and doctors.

### Collecting data and samples

In August 2023, a structured questionnaire was designed and a pilot study was conducted to assess the validity and feasibility of the questionnaire. The participants in the present study were interviewed to collect sociodemographic data, personal habits and hygiene, and clinical symptoms. Stool, blood, and saliva samples were, simultaneously, collected from every participant in sterile and prelabeled containers then delivered to the Parasitology lab, Tropical Health Department at the High Institute of Public Health. Regarding the exclusion criteria, age less than 18 or above 60 years, pregnant women, individuals on proton pump inhibitors, antibiotics, or *H. pylori* treatment, and individuals manifesting severe diarrhea (individuals having loose or watery stool more than 10 time/24 h, for a week) all were excluded from this study.

### Scoring personal hygiene parameters according to WHO guidelines

According to WHO guidelines, participants were scored for achievements of some parameters such as drinking filtered water, hand washing before eating, not eating in groups, wearing gloves and masks; the score was excellent for whom achieved 100%, good 80%, moderate 60%, limited 40%, and none for whom didn’t achieve any parameter [28]. Regarding data collection and validation, these hygiene metrics were primarily self-reported; and to ensure accuracy we implemented validation measures. These measures included cross-checking responses with direct observations where feasible, assessing consistency across multiple reports, and conducting follow-up interviews to minimize recall bias.

### Samples analysis

#### Microscopic investigation of parasitic infection

Microscopic examination is still known as the “gold standard” for the detection of intestinal parasites. PCR might be used to analyze the stool specimen in case of equivocal identification of a certain parasite. In our study we were not in need to use PCR since parasites were well identified microscopically. Although, it has been known that PCR has higher sensitivity than microscopic examination to detect intestinal parasites more accurately; but, PCR is generally not feasible in resource-poor settings, at least not in peripheral laboratories. Accordingly, microscopic examination was assigned to detect the parasitic infection [29].

Thin smears from fresh stool samples were stained after air drying by different stains including Trichrome and modified trichrome for detecting intestinal protozoa and Microsporidia, respectively [30]. Part of each stool sample was preserved in formol saline (10%) to be later concentrated using the formol-ethyl acetate sedimentation technique for microscopic detection of intestinal parasites like helminths. Then permanently stained smears were prepared from the sediment, fixed, and stained according to the standard procedures of modified Ziehl–Neelsen (MZN) technique for detecting intestinal parasites and intestinal *Coccidia* (*Cryptosporidium spp.*), respectively [31].

#### Detection of *H. pylori* antigen and antibodies (IgG and IgA)

For rapid screening of *H. pylori* antigen (Ag) in 200 fresh stool samples, *H. pylori* One Step Antigen Test Device was used for qualitative *H. pylori* detection (sensitivity, 99.5% and specificity, 97.4%); manufactured by ABON Biopharm Co, China. Positive cases obtained by rapid screening one step test have been confirmed by *H. pylori* Ag ELISA kit (sensitivity, 98.6% and specificity, > 99%), according to the instructions of the manufacturer (Creative Diagnostics, USA). Both serum and saliva samples were aliquoted, labeled, and stored at −20^◦^C until start of the immunoglobulins assay by ELISA technique. *H. pylori* IgG and IgA were assayed using ELISA kits (Creative Diagnostics, Catalog # DEIA341 for IgG (sensitivity, 96.6% and specificity, > 99%) and Catalog # DEIA342 for IgA (sensitivity, > 99% and specificity, > 99%); USA) according to the instructions of the manufacturer. All samples have been analyzed in triplicates for accuracy.

### Statistical analysis

Statistical analysis has been performed using IBM SPSS statistics, version 25.0 (IBM Corp., Armonk, NY, USA). Categorical variables have been evaluated using either Chi-square test or Fisher’s exact test. Associations and differences were considered statistically significant at P < 0.05. Cohen’s Kappa coefficient was interpreted. ≤ 0 indicates no agreement, 0.01–0.20 indicates slight agreement, 0.21–0.40 indicates fair agreement, 0.41–0.60 indicates moderate agreement, 0.61–0.80 indicates substantial agreement, and 0.81–1.00 indicates almost perfect agreement [32]. Receiver operating characteristic (ROC) analysis was performed to determine the diagnostic significance of *H. pylori* IgG and IgA levels. The ROC curve was plotted to analyze recommended cut-off values for *H. pylori* IgG and IgA. The area under the ROC curve (AUC) denotes the diagnostic performance of the *H. pylori* IgG and IgA. Agreement of the cut-off value for *H. pylori* IgG and IgA levels was expressed in sensitivity, specificity, positive predictive value, negative predictive value and accuracy. Significance of the test results is quoted as two-tailed probabilities, using student *t*-test.

## Results

A total of 130 (65%) of participants were females, drinking untreated water, having been noticed with unwashed hands before eating, and eating food together at the workplace. Approximately 60% were aged 20 years old and more residing in urban areas. Nursing students or nurses under training constituted the highest percent (41%) of participation in the present study. The majority of the participants have been noticed wearing no gloves and masks as shown in Table 1.

**Table 1:** Socio-demographic, environmental characteristics, and personal hygiene Criteria

Criteria	N	%
Gender		
Males	70	35.0
Females	130	65.0
Age in years		
< 20	82	41.0
≥ 20	118	59.0
Residence area		
Urban	118	59.0
Rural	82	41.0
Healthcare Members		
Workers	39	19.5
Employees	38	19.0
Nurses under training	82	41.0
Nurses	27	13.5
Doctors	14	7.0
Personal protective measures		
Mask		
No	195	97.5
Yes	5	2.5
Gloves		
No	200	100.0
Yes	0	0.0
Drinking water quality		
Filtered	77	38.5
Tap water	123	61.5
Washing hands prior having food		
No	121	60.5
Yes	79	39.5
Eating food Together at workplace		
No	66	33.0
Yes	134	67.0

Table 2 shows that the overall infections by intestinal parasites and *H. pylori*, of the 200 examined HCIs, were 177 cases (88.5%). Twenty-three (11.5%) cases were found to be free of infection (non-infected). *H. pylori* antigen was detected in 36 (18%) cases, as co-infection with single or multiple intestinal parasites. Also, findings revealed that *Blastocystis spp*. showed the highest rate of infection 72%, followed by *Cryptosporidium spp.*, *E. histolytica, Microsporidia*, and *Dientameba fragilis* (59.5%, 26.0%, 22.0%, and 16.5% respectively). In contrast, *Cyclospora, Giardia lamblia,* and *Isospora belli* showed lower rates of infection (5.5%, 3.5%, and 0.5%, respectively). Non-pathogenic intestinal protozoa were *Entamoeba coli* and *Iodameba bütschlii* in 14% and 3.5% of the HCIs. Also, intestinal helminths showed lower rates in *Ascaris lumbricoides* and *H. nana* (1.0% and 0.5% respectively). Concerning the multiplicity of microbial infection, double microbial infection showed the highest rate (37.5%), followed by triple or more (35.5%), and lower rate was for single infection (13.5%).

**Table 2:** Distribution of the microbial infections (including *H. pylori* co-infection) among studied healthcare individuals

Microbial Infections	Positive cases		
	No		%
Pathogenic protozoa			
*Blastocystis* spp.	144		72.0
*Cryptosporidium* spp.	119		59.5
*E. histolytica*	52		26.0
Microsporidia	44		22.0
*D. fragilis*	33		16.5
*Cyclospora*	11		5.5
*G. lamblia*	7		3.5
*I.belli*	1		0.5
Non-pathogenic protozoa			
*E. coli*	28		14.0
*I. bütschlii*	7		3.5
Helminths			
*Ascaris lumbricoides*	2		1.0
*H. nana*	1		0.5
Multiplicity of Intestinal Parasitic Infection			
Non-infected	23		11.5
Single	27		13.5
Double	65		32.5
Triple/more	85		42.5
Total Intestinal Parasitic Infections	**177**		**88.5**
Co-infection by *H. pylori* (N = 36^#^) with single or multiple intestinal parasites			
*H. pylori* and *Blastocystis* spp.		31/36	86.1
*H. pylori* and *Cryptosporidium* spp.		24/36	66.7
*H. pylori* and Microsporidia		9/36	25.0
*H. pylori* and *E. histolytica*		8/36	22.2
*H. pylori* and *G. lamblia*		5/36	13.9
*H. pylori* and *D. fragilis*		5/36	13.9
*H. pylori* and *E. coli*		2/36	5.6
*H. pylori* and *I. bütschlii*		1/36	2.8
*H. pylori* and *A. lumbricoides*		1/36	2.8
Total Co-infections		**36**	**18**

^**#**^: It means 36 cases show co-infection by *H. pylori* with single or multiple intestinal parasites

Table 3 shows the multiplicity of microbial infection (including *H. pylori* infection) among the HCIs. Healthcare individuals were categorized according to job type. It has been noticed that the highest rates of infection were detected among workers and nurses (100% for each), followed by employees and nurses under training (students) (97.4% and 81.7% respectively), in contrast, the lowest rate was detected among doctors (50%). Concerning the multiplicity of infection among HCIs, the highest rate of triple or more was detected among the workers (76.9%), followed by nurses, employees, nurses under training, and doctors (59.3%, 44.7%, 25.6%, and 7.1%, respectively). The highest double infection rate was observed among nurses under training (40.2%), followed by employees, nurses, doctors, and workers (34.2%, 33.3%, 21.4%, and 17.9%, respectively). Finally, the highest rate of single infection was detected among doctors (21.4%), followed by employees, nurses under training, nurses, and workers (18.4%, 15.9%, 7.4%, and 5.1%, respectively).

**Table 3:** Multiplicity of the microbial infections (including *H. pylori* co-infection) among studied healthcare Individuals

Studied heath care members	Total examined (n = 200)	^#^Total infected (n = 177)	^#^Multiplicity of microbial infection			
			Non-infected No. (%) (n = 23)	Single No. (%) (n = 27)	Double No. (%) (n = 65)	Triple/more No. (%) (n = 85)
Workers	39	39 (100)	0 (0.0%)	2 (5.1%)	7 (17.9%)	30 (76.9%)
Employees	38	37 (97.4)	1 (2.6%)	7 (18.4%)	13 (34.2%)	17 (44.7%)
Nurses under training	82	67 (81.7)	15 (18.3%)	13 (15.9%)	33 (40.2%)	21 (25.6%)
Nurses	27	27 (100)	0 (0.0%)	2 (7.4%)	9 (33.3%)	16 (59.3%)
Doctors	14	7 (50)	7 (50.0%)	3 (21.4%)	3 (21.4%)	1 (7.1%)

^**#**^: Including the 36 cases co-infected by *H. pylori*

Table 4 demonstrates the multiplicity of the microbial infection among HCIs in relation to the score of personal hygiene. The highest rate of microbial infection was detected among those who have zero score of personal hygiene (100% with infection(s)), followed by those with limited score (91%), and 55% among those with moderate score, meanwhile, no infection was detected among those with a good score of personal hygiene. Ungraded HCIs showed a high statistically significant difference (***P* < 0.001**) of infection with triple or more microbial infection as compared to those infected with single or double infection (67.7% vs. 4.6% and 27.7%, respectively). In contrast, HCIs with limited scores showed high double infection rate with no significant difference (***P* > 0.05**) as compared to those infected with single and triple or more infection (38.7% vs. 15.3% and 36.9%, respectively). HCIs with moderated scores showed high single infection rate, followed by double infection rate (35.0% and 20.0%, respectively) with statistically significant difference (***P* < 0.001**) compared with HCIs who had no infection (45.0%). HCIs with a good score of personal hygiene showed zero infection (100% with no any infection).

**Table 4:** Multiplicity of the microbial infections (including *H. pylori* infection) among studied healthcare Individuals in relation to the score of personal hygiene

Score of Personal Hygiene	Total Examined	^**#**^Multiplicity of Microbial Infection				χ^2^	p	Total Microbial Infection
		Non-infected No. (%) (n = 23)	Single No. (%) (n = 27)	Double No. (%) (n = 65)	Triple/more No. (%) (n = 85)			
None	65	0 (0.0%)	3 (4.6%)	18 (27.7%)	44 (67.7%)	31.769*	< 0.001*	65 (100%)
Limited	111	10 (9.0%)	17 (15.3%)	43 (38.7%)	41 (36.9%)	6.758	0.080	101 (91%)
Moderate	20	9 (45.0%)	7 (35.0%)	4 (20.0%)	0 (0.0%)	35.785*	^MC^p < 0.001*	11 (55%)
Good	4	4 (100.0%)	0 (0.0%)	0 (0.0%)	0 (0.0%)	14.326*	^MC^p < 0.001*	0 (0.0)

χ^2^ Chi square test, *MC* Monte Carlo

p p value for Relation between score of personal hygiene and multiplicity of microbial infection

* Statistically significant at p ≤ 0.05

^**#**^: Including the 36 cases co-infected by *H. pylori*

Regarding risk factors, males exhibited higher risks for microbial infection compared to females (91.4% vs. 89.2%, respectively; i.e. males at 1.287-fold risk compared to females), yet the difference wasn’t statistically significant (***P* > 0.05**). HCIs aged ≥ 20 years old were three times at risk for microbial infection as compared to those < 20 years old (94.1% vs. 84.1%) with a statistically significant difference (***P* < 0.05**). In contrast, HCIs who resident to rural areas were at insignificant risk factor compared to those who resident urban areas [(1.705-fold), (***P* > 0.05**)]. Workers, employees, and nurses showed the highest rate of infection (100.0%), and nurses under training exhibited a protective risk factor (OR = 0.335; 0.127–0.880), then was observed among doctors (OR = 0.075; 0.023–0.247). According to personal protective measures, HCIs, who wearing no gloves and masks, were at risk for microbial infection as compared to those who wear gloves and masks (92.3% and 90%, respectively). The present findings showed that untreated drinking water, unwashed hands before eating, and eating together at the workplace exhibited a highly statistically significant differences risk factor among HCIs [(11.33-fold), (38.0-fold), and (5.74-fold), respectively; with (***P* < 0.001**)]. Contrarily, contact with animals (e.g. pets in general) showed insignificant risk factor for HCIs (OR = 1.32; 0.457–3.812); that could be owing to the diversity of animal species and varying frequency of contact with pets (Table 5).

**Table 5:** Microbial Infection and risk factors among the studied sample according to socio demographic data

Criteria	Total examined	Microbial Infection N (%)	OR (LL – UL 95%C.I)	P
Gender				
Males	70	64 (91.4)	1.287 (0.472 – 3.513)	0.622
Females	130	116 (89.2)	1.000	
Age in years				
< 20	82	69 (84.1)	1.000	0.026^*^
≥ 20	118	111 (94.1)	2.988 (1.136 – 7.855)	
Residence area				
Urban	118	104 (88.1)	1.000	0.296
Rural	82	76 (92.7)	1.705 (0.627 – 4.640)	
Healthcare Members				
Workers	39	39 (100)	–	0.998
Employees	38	38 (100)	–	0.998
Nurses under training	82	69 (84.1)	0.335 (0.127 – 0.880)	0.026^*^
Nurses	27	27 (100)	–	0.998
Doctors	14	7 (50)	0.075 (0.023 – 0.247)	< 0.001^*^
Personal protective measures				
Mask				
No	195	180 (92.3)	1.000	0.999
Yes	5	0 (0.0)	–	
Gloves				
No	200	180 (90)	1.000	–
Yes	0	0 (0.0)	–	
Drinking water quality				
Filtered	77	60 (77.9)	1.000	< 0.001^*^
*Tap water	123	120 (97.6)	11.333 (3.196 – 40.191)	
Washing hands prior having food				
No	121	120 (99.2)	38.00 (4.968 – 290.675)	< 0.001^*^
Yes	79	60 (75.9)	1.000	
Eating food Together at Workplace				
No	66	52 (78.8)	1.000	0.001^*^
Yes	134	128 (95.5)	5.744 (2.094 – 15.757)	
Contact with animals				
No	140	125 (89.3)	1.000	0.608
Yes	60	55 (91.7)	1.320 (0.457 – 3.812)	

*OR* Odd`s ratio, *C.I* Confidence interval, *LL* Lower limit, *UL* Upper Limit

*: Statistically significant at p ≤ 0.05

Table 6 shows equal rates of *H. pylori* IgG and IgA levels in both serum and salivary samples (6.5% and 1.0%, respectively), exhibiting statistically significant difference for quantitative IgG in serum samples as compared to that detected in salivary samples ***P* < 0.001** [(8.93 ± 35.23) vs. (5.95 ± 20.85), respectively] and the same observed in IgA, ***P* < 0.001** [(0.146 ± 0.117) vs. (0.136 ± 0.112), respectively].

**Table 6:** Comparison between ELISA techniques in detecting serum and saliva IgG and IgA of *H. pylori*

Immunoglobulins analysis	Serum (n = 200)	Saliva (n = 200)	Test of Sig	p
Qualitative analysis				
IgG	13 (6.5%)	13 (6.5%)	–	^McN^p = 1.000
IgA	2 (1%)	2 (1%)	–	^McN^p = 1.000
Quantitative analysis				
IgG				
Min. – Max	1.0 – 300.0	1.0 – 212.0	Z = 4.848*	< 0.001*
Mean ± SD	8.93 ± 35.23	5.95 ± 20.85		
IgA				
Min. – Max	0.1 – 1.0	0.1 – 1.0	Z = 3.443*	0.001*
Mean ± SD	0.146 ± 0.117	0.136 ± 0.112		

*SD* Standard deviation, *McN* McNemar test, *Z* Wilcoxon signed ranks test

p: p value for comparing between **Serum** and **Saliva**

*: Statistically significant at p ≤ 0.05

**Fig. 1:**
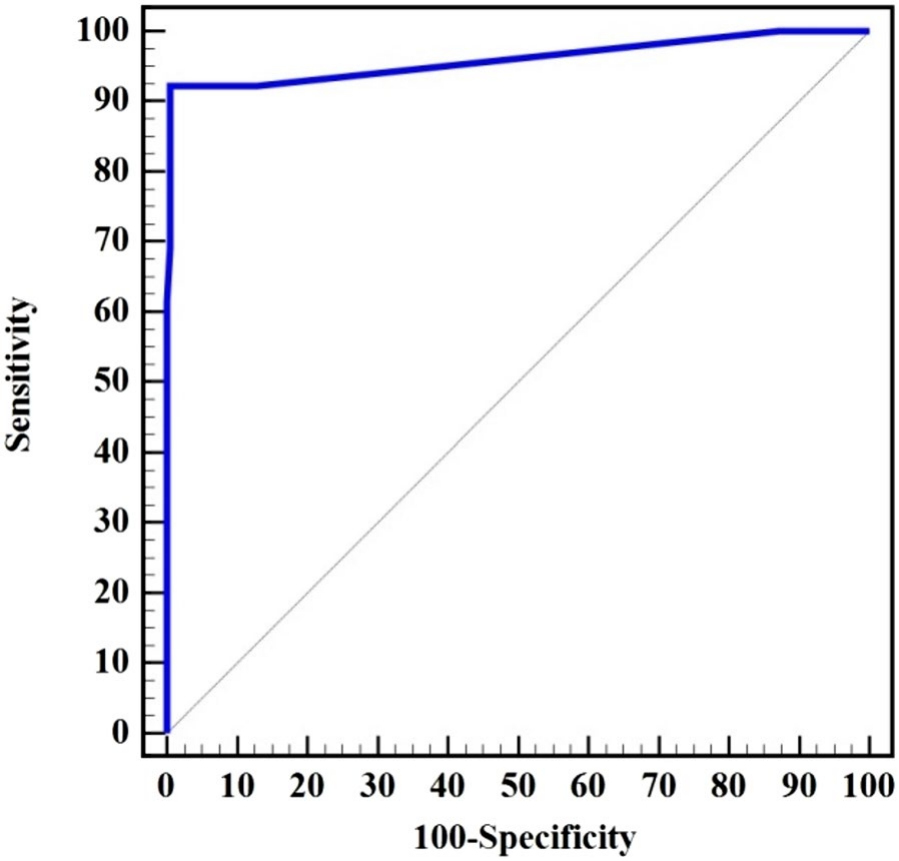
ROC curve for *H. pylori* IgG detection in Saliva compared to Serum (n = 13 vs. 187)

Our findings revealed that 12 cases were reported positive for *H. pylori* IgG by ELISA in both serum and saliva samples (Fig. 1). Moreover, two extra cases were found to be positive for *H. pylori* IgG (i.e. total number of IgG positive cases = 14); one tested positive in serum but negative in saliva (i.e. 13 cases were IgG positive in serum) and the other one tested negative in serum but positive in saliva (i.e. 13 cases were IgG positive in saliva). In general, 186 cases were found to be completely negative for *H. pylori* IgG either in serum or salivary samples. Cohen’s Kappa coefficient (K) was 0.918, indicating perfect agreement between serum and salivary ELISA techniques Table 7. ELISA of salivary samples for *H. pylori* IgG detection had a sensitivity of 92.3% when compared to ELISA of serum samples for IgG detection, and the specificity was 99.5%. Additionally, PPV, NPV, and accuracy were 92.3%, 99.5%, and 99.0%, respectively, the AUC was 0.96, and the cut off > 5 (Table 7 and Fig. 1).

**Table 7:** Diagnostic performance of ELISA for *H. pylori* IgG detection in Saliva compared to Serum

IgG	Serum		k	Sensitivity	Specificity	PPV	NPV	Accuracy	AUC	95% C.I	Cut off^#^
	Negative (n = 187)	Positive (n = 13)									
	No. (%)	No. (%)									
Saliva											
Negative	186 (99.5%)	1 (7.7%)	0.918	92.3	99.5	92.3	99.5	99.0	0.960	0.881 – 1.000	> 5
Positive	1 (0.5%)	12 (92.3%)									
** χ**^**2**^** (**^**FE**^**p)**	168.445^*^ (< 0.001^*^)										

*PPV* Positive predictive value, *NPV* Negative predictive value, *AUC* Area Under a Curve, *CI* Confidence Intervals

χ^2^ Chi square test, k Cohen’s Kappa coefficient

p: p value for association between different categories

*: Statistically significant at p ≤ 0.05

^#^Cut off was choose according to Youden index

**Fig. 2:**
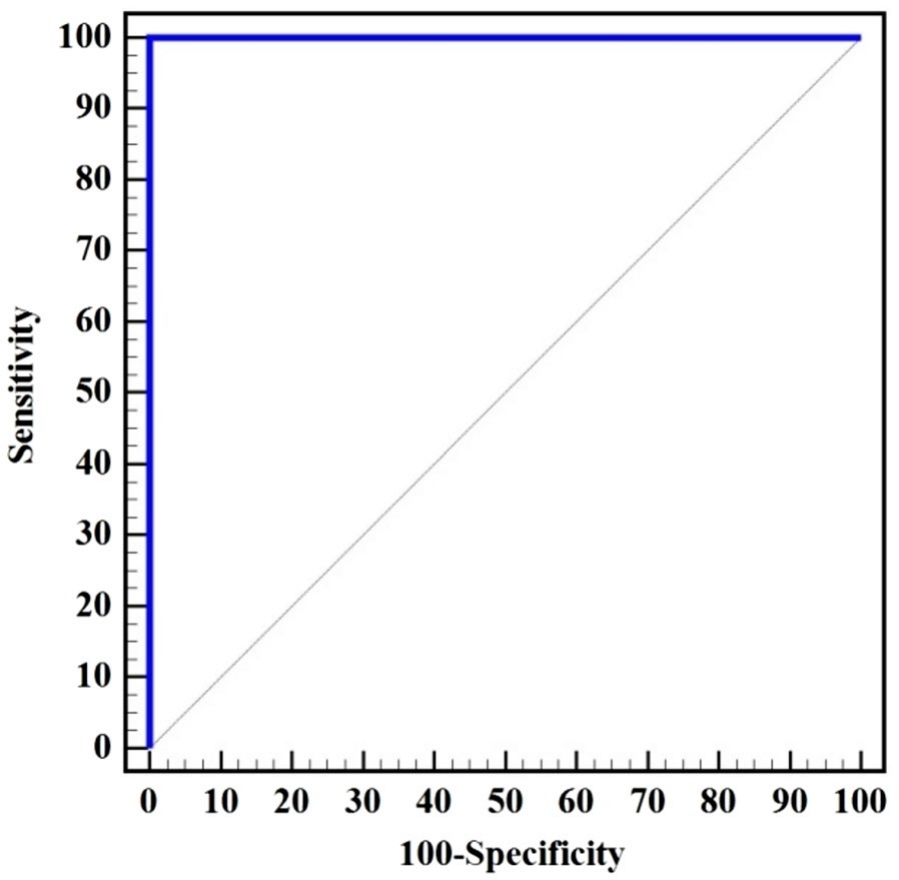
ROC curve for *H. pylori* IgA detection in Saliva compared to Serum (n = 2 vs. 198)

As regards ELISA detection of *H. pylori* IgA, 2 cases were found to be positive in both serum and salivary samples while 198 cases were negatives in both serum and saliva samples (Fig. 2). No cases were found to be positive for IgA in saliva while it was negative in serum and vice versa. Cohen’s Kappa coefficient (K) was 1.0 indicating excellent agreement between serum and salivary ELISA techniques. ELISA technique for detecting *H. pylori* IgA in saliva had a sensitivity of 100.0% and a remarkable specificity of 100.0% compared to ELISA technique for detecting IgA in serum. The PPV, NPV, and accuracy were 100.0% each, the AUC was 1.0, and the cut off > 0.6 (Table 8 and Fig. 2).

**Table 8:** Diagnostic performance of ELISA for *H. pylori* IgA detection in Saliva compared to Serum

IgA	Serum		k	Sensitivity	Specificity	PPV	NPV	Accuracy	AUC	95% C.I	Cut off^#^
	Negative (n = 198)	Positive (n = 2)									
	No. (%)	No. (%)									
Saliva											
Negative	198 (100%)	0 (0%)	1.000	100.0	100.0	100.0	100.0	100.0	1.000	1.000 – 1.000	> 0.6
Positive	0 (0%)	2 (100%)									
** χ**^**2**^** (**^**FE**^**p)**	200.00* (< 0.001*)										

*PPV* positive predictive value, *NPV* negative predictive value, *AUC* area under a curve, *CI* confidence intervals

χ^2^ Chi square test, k Cohen’s Kappa coefficient

p: p value for association between different categories

*: Statistically significant at p ≤ 0.05

^#^Cut off was choose according to Youden index

## Discussion

Healthcare individuals encounter many different types of hazards in their workplaces; some of these hazards are committed by individuals themselves. That’s owing to inappropriate application of regulations for wearing personal protective equipment, personal hygiene, and socio-economic lifestyle. Accordingly, this study has been performed to highlight the inappropriate behaviors of healthcare individuals toward their personal safety for mitigation of the risk of gastrointestinal microbial infections by improving personal hygiene. Also, this study is the first cross sectional study that stratified the healthcare individuals into; workers, employees, nursing students, nurses, and doctors to correlate personal hygiene and socio-economic lifestyle with gastrointestinal microbial infections caused either by parasites or *H. pylori*. Meanwhile, this study compared the levels of *H. pylori* antibodies (IgG and IgA) in serum and saliva samples trying to find a surrogate non-invasive method for laboratory detection of *H. pylori* antibodies.

Hand hygiene remains a common factor of healthcare-associated infections [33]. Transmission of pathogens between the healthcare environment, healthcare workers, and patients, is facilitated by contaminated hands [34].

Healthcare individuals, who provide patient care, work in research and clinical laboratories are at risk of becoming infected with parasites like other microbial infections which may occur through accidental exposures. Moreover, healthcare workers infected with parasites or other microbial infections may infect patients; mainly critical care patients, who are highly susceptible to various infections due to immunocompromised state, and also may infect their colleagues [35].

Current study recruited 200 healthcare individuals; more than 60% of them were females. Nursing students or nurses under training constituted the highest percent (41%) of participants. Majority of participants are drinking untreated water, have been noticed with unwashed hands before eating, eat food together, and wear no gloves and masks. The study revealed a high rate of microbial infections and intestinal parasitic infections among healthcare individuals. Some cases have single, double, triple microbial infection or even more. Only 36 cases (18%) of participants were co-infected by *H. pylori*, as they tested positive for *H. pylori* Ag. The highest rates of infection were detected among workers and nurses (100% for each), followed by employees and nurses under training (students), in contrast, the lowest rate of infection was detected among doctors. Nurses and workers showed highest rates in triple microbial infection while doctors showed the highest rate in single microbial infection. It was noticed that the highest rate of microbial infection was detected among those who have zero score of personal hygiene (100% with infection(s)), followed by those with limited scores, and moderate scores; meanwhile no infection was detected among those with a good score of personal hygiene. In contrast, HCIs with limited scores showed high double infection rate with no significant difference as compared to those infected with single and triple or more infection. HCIs with moderated scores showed high single infection rate, followed by double infection rate with statistically significant difference compared with HCIs who had no infection. HCIs with a good score of personal hygiene showed zero infection (100% with no any infection). A remarkable note is that there was not any significant difference regarding the risk factor of microbial infection between males and females of HCIs. Healthcare individuals aged ≥ 20 years old were, significantly, 3 times at risk for microbial infection as compared to those < 20 years old. In contrast, HCIs who reside in rural areas were at an insignificant risk factor compared to those who resided in urban areas. Workers, employees, and nurses showed the highest rate of infection, and nurses under training (nursing students) exhibited a protective risk factor, then was observed among doctors. According to personal protective measures, HCIs, who have been noticed wearing no gloves and masks, were at risk for microbial infection as compared to those who wear gloves and masks. The present findings showed that untreated drinking water, unwashed hands before eating and eating together at the workplace exhibited an increased incidence of being at high risk for microbial infections. As a consequence, one can deduce that it is absolutely owing to the differences in socio-economic lifestyle and personal hygiene among each category of healthcare individuals. Unintentionally, healthcare individuals (HCIs) are transmitting pathogenic microorganisms from one patient to another on their hands, consequently; vulnerable patients develop the infections [36]. Furthermore, wearing sterile gloves and masks does not achieve a complete reduction of microbial infection and does not replace hand hygiene; that is because of sharing the equipment and lack of places to work or put equipment and paperwork [37].

It has been reported that hand washing has been identified as the most important single behavior change that healthcare workers can make for infection control [36, 38]. The strict practice of hand washing strategies in hospitals have been observed to be weak, with multiple research studies reporting that globally, in hospitals a regular hand wash by healthcare workers often does not exceed 40% [39, 40]. Nurses are among the healthcare individuals who spend most of their work hours in contact with patients [41]. Nursing students are known to have a low level of hand wash knowledge and practice, often, due to the influence of personal and administrative issues [42].

Non-compliance with the guidelines of hand wash knowledge and practice is regarding a global public health issue which requires more effective policies, surveillance, and conducting more research investigations [40].

Among microbial infections, the most redundant infection is *Helicobacter pylori* (*H. pylori*) infection which is chronic and usually acquired in childhood. H. pylori infect 50% of population worldwide; whom are influenced by socioeconomic status, sanitation, regions, and age. It was reported that Africa had the highest prevalence of H. pylori infection (70.1%). On the contrary, industrialized nations showed a lower *H. pylori* prevalence as for instance the United States (35.6%), Japan (51.7%) and China (55.8%) because of advanced healthcare services and good sanitation infrastructure [43].

Several laboratory diagnostic tests are available for detecting *H. pylori* infection, including conventional noninvasive methods such as the Urea Breath Test (UBT) and stool antigen tests, as well as serological tests that detect *H. pylori* antibodies in the blood, which are considered invasive due to the need for blood withdrawal. However, UBT has certain limitations, requiring the discontinuation of proton pump inhibitors (PPIs) for at least two weeks and antibiotics or bismuth compounds for at least four weeks, as these can reduce *H. pylori* load and affect test accuracy [44–46]. Similarly, stool antigen detection has limitations, primarily due to the low density of *H. pylori* in the stomach and the low antigen load in stool, leading to false-negative results. Factors such as the use of bismuth, PPIs, or antimicrobials, unformed or watery stool samples, and the timing of sample collection post-eradication can impact results [47, 48]. Additionally, temperature and the time between stool collection and testing can influence accuracy. [49]. Beyond conventional methods, molecular techniques such as PCR can be used to detect *H. pylori* in saliva and stool [50, 51]. However, PCR-based diagnosis has its own challenges, including the selection of target genes for primer design, the availability of commercial DNA extraction kits for saliva and stool, and the potential for false-positive results. [52]. The majority of serological tests demonstrate a specificity exceeding 90%, while their sensitivity ranges from 60 to 90%. However, ELISA-based serological methods currently lack consistent reliability. Since serology primarily detects past infections rather than active ones, it is not suitable for assessing eradication success. Considering regional differences in infection prevalence, bacterial load, and strain distribution, the development and validation of locally tailored serology kits would be more effective. Although new rapid near-patient tests are undergoing evaluation, they may achieve the required accuracy standards in the future. [53, 54].

This study aimed to compare levels of *H. pylori* antibodies (IgG and IgA) in saliva (as a noninvasive test) and in serum rather than tracing the significance of immunoglobulins detection; that’s for finding a surrogate non-invasive method for laboratory detection of *H. pylori* antibodies. Serum immunoglobulins’ response to *H. pylori* infection is an important determinant of gastric mucosal damage. IgA is the predominant immunoglobulin in mucosal secretions of the gastrointestinal tract. Elevated levels of *H. pylori* IgA may indicate an active or recent infection. IgG is an important marker for detecting past exposure to *H. pylori*. IgA and IgG type of antibodies may remain high for months or years unless the infection is treated. After eradication of *H. pylori*, IgA levels decrease but IgG does not disappear [17, 18]. Salivary IgG is secreted during the serum immune response, and the levels parallel that of circulatory IgG levels [19].

Results showed that 12 cases that were reported positive for IgG by ELISA of salivary samples were also positive by the ELISA of serum samples. On the other hand, one case which was IgG negative in salivary samples was actually IgG positive in serum samples. That could be owing to the course of *H. pylori* infection for this case, in other meaning the IgG production begins in circulating plasma during early infection. Also, it is known that salivary IgG is initially derived from serum through gingival clefts by passive diffusion, although some is locally produced [55]. On the contrary, one case of the IgG negative serum samples was tested IgG positive in salivary samples. That case could be considered as false positive due to some reasons such as *H. pylori*-related periodontal disease which seems to be the main cause in our case, or actively bleeding gums which is not applicable in our case due to the absence of serum IgG [56]. 186 cases were IgG negative in both serum and salivary samples. Cohen’s Kappa coefficient (K) indicated a perfect agreement between serum and salivary ELISA techniques. As regards ELISA detection of IgA, two cases were found to be positive in both serum and salivary samples that could reflect an active or recent infection [17]. While 198 cases were tested negative in both serum and saliva. No cases were found to be positive for IgA in saliva while it was negative in serum and vice versa. Also, Cohen’s Kappa coefficient (K) indicated an excellent agreement between serum and salivary ELISA techniques. It was reported that IgG antibodies show quantitatively equal reactivity in serum and saliva; and, practically, any saliva-based IgG assay (classical serology) could be implemented as serum-based assay with no difference [57]. Similarly, IgA antibodies show no significant differences in their levels between serum and saliva [58].

One confusing issue was noticed while analyzing samples for *H. pylori* antigen and antibodies, which is 36 cases found to be positive for *H. pylori* antigen in stool, while 14 cases positive for IgG and 2 cases positive for IgA in serum and saliva. The interpretation for such confusion, considering the co-infection by other intestinal parasites, is to recall a study that showed 3 of 16 (18.75%) rapid urease test-negative patients were tested positive for *H. pylori* antigen in stool. This positivity might be owing to a cross-reactivity with an antigen from *H. pylori* species or other microorganisms of the intestinal flora; therefore, the results are considered as false positives [59]. Another study showed the sensitivity and specificity of immunological techniques for detecting *H. pylori* infection ranging from 80 to 90%. However, this observation should be reconsidered due to variations in individual immune responses, the timing of infection exposure, nutritional status, and potential interference from antigenically related bacteria [60]. Additionally, IgA levels can fluctuate based on the stage of infection and immune response, sometimes leading to lower sensitivity and specificity, as well as reduced accuracy in detecting active infections. Moreover, while IgG signifies past infection, IgA indicates an active or recent infection. The only shared characteristic between the two is their prolonged high levels in untreated infections. In this study, the commonality between IgA and IgG is not related to the infection timeline but rather to their ability to detect infections that have remained untreated for an extended period [53, 54].

*In conclusion*, this study found that the inappropriate personal hygiene and socio-economic lifestyle of healthcare individuals are directly correlated with the risk of gastrointestinal microbial infections. Moreover, the current study compared the levels of *H. pylori* antibodies (IgG and IgA) in serum and saliva samples, and then concluded that saliva immunoglobulins could be used as a non-invasive surrogate of serum immunoglobulins for the detection of *H. pylori* infection. One limitation of the study is that only a single random stool sample from each participant was used for microscopic examination of intestinal parasites, rather than three consecutive samples. Nevertheless, larger scale investigations across different healthcare settings are recommended to validate our findings, especially re-evaluation of salivary immunoglobulins as a potential alternative for serum. Moreover, a longitudinal study is needed for assessment of infection persistence and reinfection rates among healthcare individuals; this is after informing them about risk of infection and how to avoid it.

## Data Availability

No datasets were generated or analysed during the current study.

## Abbreviations

Ag: Antigen
AUC: Area under the curve
K: Cohen’s Kappa coefficient
*D. fragilis*: *Dientamoeba fragilis*
*E. coli*: *Entamoeba coli*
*E. histolytica*: *Entamoeba histolytica*
ELISA: Enzyme-linked immunosorbent assay
*G. lamblia*: *Giardia lamblia*
HCIs: Healthcare individuals
*H. pylori*: *Helicobacter pylori*
*H. nana*: *Hymenolepis nana*
Ig: Immunoglobulin
IPI: Intestinal parasitic infection
*I. bütschlii*: *Iodameba bütschlii*
NPV: Negative predictive values
NET: Neutrophil extracellular trap
PPV: Positive predictive values
PPIs: Proton pump inhibitors
ROC: Receiver operating characteristic curve
UBT: Urea Breath Test
